# Hydrogen Sulfide Ameliorates Lung Ischemia-Reperfusion Injury Through SIRT1 Signaling Pathway in Type 2 Diabetic Rats

**DOI:** 10.3389/fphys.2020.00596

**Published:** 2020-06-30

**Authors:** Tao Jiang, Weiwei Yang, Hongli Zhang, Zhiqiang Song, Tianhua Liu, Xiangqi Lv

**Affiliations:** ^1^Department of Anesthesiology (Hei Long Jiang Province Key Lab of Research on Anesthesiology and Critical Care Medicine), The Second Affiliated Hospital, Harbin Medical University, Harbin, China; ^2^Department of Pathology, Harbin Medical University, Harbin, China; ^3^Department of Ophthalmology, Daqing Fifth Hospital, Daqing, China; ^4^Department of Geriatrics, The Second Affiliated Hospital, Harbin Medical University, Harbin, China

**Keywords:** hydrogen sulfide, SIRT1, lung ischemia-reperfusion injury, type 2 diabetes mellitus, Oxidative Stress

## Abstract

Lung ischemia-reperfusion (IR) injury remains a significant factor for the early mortality of lung transplantations. Diabetes mellitus (DM) is an independent risk factor for 5-year mortality following lung transplantation. Our previous study showed that DM aggravated lung IR injury and that oxidative stress played a key role in this process. Previously, we demonstrated that hydrogen sulfide (H_2_S) protected against diabetic lung IR injury by suppressing oxidative damage. This study aimed to examine the mechanism by which H_2_S affects diabetic lung IR injury. High-fat-diet-fed streptozotocin-induced type 2 diabetic rats were exposed to GYY4137, a slow-releasing H_2_S donor with or without administration of EX527 (a SIRT1 inhibitor), and then subjected to a surgical model of IR injury of the lung. Lung function, oxidative stress, cell apoptosis, and inflammation were assessed. We found that impairment of lung SIRT1 signaling under type 2 diabetic conditions was further exacerbated by IR injury. GYY4137 treatment markedly activated SIRT1 signaling and ameliorated lung IR injury in type 2 DM animals by improving lung functional recovery, diminishing oxidative damage, reducing inflammation, and suppressing cell apoptosis. However, these effects were largely compromised by EX527. Additionally, treatment with GYY4137 significantly activated the Nrf2/HO-1 antioxidant signaling pathway and increased eNOS phosphorylation. However, these effects were largely abolished by EX527. Together, our results indicate that GYY4137 treatment effectively attenuated lung IR injury under type 2 diabetic conditions via activation of lung SIRT1 signaling. SIRT1 activation upregulated Nrf2/HO-1 and activated the eNOS-mediated antioxidant signaling pathway, thus reducing cell apoptosis and inflammation and eventually preserving lung function.

## Introduction

The prevalence of diabetes is dramatically increasing across all age groups around the world (Chen et al., 2011). There is now increasing evidence indicating that the lung is one of the target organs for diabetic injury in diabetes mellitus (DM) patients (Kuitert, 2008; Pitocco et al., 2012). Diabetes mellitus, especially type 2 DM, is an independent risk factor for morbidity and mortality at both 1 and 5 years following lung transplantation (Christie et al., 2010). Several studies have demonstrated that peri-operative hyperglycemia is a critical factor leading to the poor survival in lung transplant recipients (Hackman et al., 2014). Lung ischemia-reperfusion (IR) injury can contribute to acute graft failure, which is the major risk factor for mortality in the early period of lung transplantation (De Perrot et al., 2003). Under the diabetic condition, sustained hyperglycemia dramatically induces reactive oxygen species (ROS) overproduction and impairs antioxidant defenses, thereby aggravating oxidative stress and causing cell necrosis and apoptosis (Evans et al., 2002; Zheng et al., 2017). Furthermore, ROS are a stimulatory signal of Nuclear factor-kappa B (NF-κB) activation, which aggravates inflammatory response by upregulating tumor necrosis factor-α (TNF-α) (Donath and Shoelson, 2011). Oxidative stress and inflammation play key roles in the processes of apoptosis, which may account for the poor outcome in diabetic patients following lung transplantation (Hackman et al., 2014). We have demonstrated that DM aggravated lung IR insult and that enhanced oxidative stress played a crucial role in diabetic lung IR insult (Song et al., 2017; Jiang et al., 2019). Therefore, novel strategies ameliorating diabetic lung IR injury may derive from studies scavenging free radicals or targeting the sources of ROS.

Silent information regulator 1 (SIRT1) is a nicotinamide adenine dinucleotide (NAD^+^)-dependent deacetylase that participates in multiple cellular functions and exerts influence in tissue injury and repair (Winnik et al., 2015). By deacetylating target proteins such as endothelial nitric oxide synthase (eNOS), erythroid 2-related factor 2 (Nrf2), PGC-1α, and FOXOs, SIRT1 regulates cellular homeostasis (Motta et al., 2004; Mattagajasingh et al., 2007). Growing evidence has shown that SIRT1 signaling is responsive to the oxidative stress and inflammation that is caused by a variety of acute lung injuries (Guo et al., 2015; Sun et al., 2017a). Silent information regulator 1 also plays a protective role in lung IR injury (Liu et al., 2016). Notably, emerging data suggested that SIRT1 is a promising therapeutic target for the treatment of type 2 DM by regulating insulin sensitivity and glucose-lipid homeostasis in both animal studies and clinical research (Kitada and Koya, 2013). Recent research has focused on the role of SIRT1 in the treatment of IR injury in the diabetic state.

Hydrogen sulfide (H_2_S) is a new gasotransmitter with a wide range of physiological functions (Kasparek et al., 2012; Wallace and Wang, 2015). Circulating levels of H_2_S are decreased in both patients with type 2 DM and diabetic animal models, as hyperglycemic cells consume and oxidize H_2_S, which contributes to the development of diabetic complications (Jain et al., 2010). Our previous study showed that H_2_S attenuated diabetic lung IR injury, although the potential mechanisms remain unexplored (Jiang et al., 2019). Moreover, H_2_S is able to upregulate SIRT1 to protect against oxidative stress in cardiomyocytes (Wu et al., 2015). However, whether the SIRT1 signaling pathway is involved in H_2_S’s protective effect on diabetic lung IR injury is still unknown.

Here, we employed GYY4137 as a slow-releasing H_2_S donor drug to evaluate whether SIRT1 signaling plays a regulatory role in the protective actions of H_2_S treatment against diabetic lung IR injury and observed the signaling pathways involved in the ameliorative effect of H_2_S on oxidative stress.

## Materials and Methods

### Animals

Pathogen-free male Sprague Dawley (SD) rats weighing 200–220 g were purchased from the Animal Experiment Center of Harbin Medical University (Harbin, Heilongjiang, China). The animals were allowed free access to food and water and maintained at 22–24°C with a light/dark cycle (12/12 h). This study was carried out in accordance with the National Institutes of Health Guidelines for the Care and Use of Laboratory Animals (NIH Publication No. 85–23, revised, 1996). All study protocols were approved by the Institutional Animal Care and Use Committee at Harbin Medical University.

### Type 2 Diabetic Rat Model

The type 2 diabetic rat model was established as described previously (Jiang et al., 2019). Briefly, rats were fed with high-fat food containing (2.5% cholesterol, 5% sesame oil, 15% lard, 20% sucrose, and 57.5% normal chow) for 6 weeks. After that, streptozotocin (STZ, 35 mg/kg) was intraperitoneally injected. Then, rats were continuously fed with high-fat food, and diabetes was defined as existing in rats with fasting plasma glucose above 11.1 mmol/L 72 h after STZ injection. The standard laboratory chow-fed rats were studied as the non-diabetic controls. We measured the glucose tolerance of various groups by conducting the intraperitoneal glucose tolerance test (IPGTT) and the oral glucose tolerance test (OGTT) to confirm the success of the diabetic animal model. Glucose was administered at a dose of 2 g/kg by intraperitoneal injection or gastric lavage to 12-h-fasted rats. The blood glucose level was tested at 0 (before glucose load), 30, 60, 90, and 120 min after glucose load.

### Rat Lung IR Model

The lung IR model was performed as described previously (Jiang et al., 2019). Briefly, under sodium pentobarbital anesthesia (40 mg/kg), the rats were intubated with a 12-gauge through a tracheostomy and ventilated with a tidal volume of 10 ml/kg, which led to an inspired oxygen fraction (FiO_2_) of 40% (40% oxygen and 60% nitrogen) at a positive end-expiratory pressure of 2 cm H_2_O. The breathing rate was adjusted to maintain arterial carbon dioxide tension (PaCO_2_) at 35 to 45 mmHg. A 24-gauge catheter was inserted into the right femoral artery for pressure monitoring (Datex, Helsinki, Finland) and arterial blood sampling analysis (Bayer, Medfield, MA, United States), and the right femoral vein was also cannulated for drug administration. After thoracotomy, the left lung hilum was clamped with a non-clash microclip 5 min after the administration of heparin (50 IU/animal) at the end of expiration. Subsequently, the tidal volume was adjusted to 6 ml/kg during clamping. The left lung hilum was clamped for 90 min and released, and reperfusion proceeded for 4 h while the tidal volume was restored to 10 ml/kg. All rats were positioned on a heating pad to maintain body temperature. Pipecuronium bromide (0.4 mg/kg/h) was used to maintain muscle relaxation, and anesthesia was maintained with sodium pentobarbital. Rats in the sham groups underwent the same procedure except for the left lung hilum occlusion.

### Experimental Groups

The animals were randomly assigned to the following groups (*n* = 8 in each group): sham group (Con + Sham), lung IR group (Con + IR), DM + sham group (DM + Sham), DM + IR group (DM + IR), DM + IR + GYY4137 group (DM + IR + H), DM + IR + GYY4137 + EX527 group (DM + IR + H + E), and DM + IR + EX527 group (DM + IR + E). GYY4137 (133 μmol/kg, dissolved in 1.0 ml of sterile normal saline) was intraperitoneally injected 1 h prior to the surgery. EX527 (the inhibitor of SIRT1 signaling, 5 mg/kg/day) was dissolved in dimethyl sulfoxide (DMSO) and then diluted to the final concentration with sterile saline (the final DMSO concentration was 1%). EX527 was intraperitoneally injected for 3 days before the surgery and once 20 min before the reperfusion. The doses of GYY4137 and EX527 were selected on the basis of our previous studies (Yu et al., 2017; Jiang et al., 2019).

### Histological Analysis

The lung tissues were fixed in paraformaldehyde, embedded in paraffin, sectioned (5-μm thickness) and stained with hematoxylin and eosin. Injury morphology was assessed under a light microscope in the following respects: (1) airway epithelial cell damage, (2) hyaline membrane formation, (3) interstitial edema, (4) hemorrhage, and (5) neutrophil infiltration. The degree of lung injury was scored on a semiquantitative scale of 0–4 as follows: normal = 0, minimal change = 1, mild change = 2, moderate change = 3, and severe change = 4.

### Measurement of the Static Compliance of the Lungs and Wet/Dry Lung Weight Ratio

As previously described, median sternotomies were performed immediately after sacrifice, and the animals were connected to an apparatus to measure the static pressure–volume (P–V) curves of the lungs (Jiang et al., 2019). At the time of rat death, the upper section of the lung was placed in an oven at 80°C for 72 h, and the wet weight-to-dry weight ratio (W/D) was measured.

### Blood Gas Analysis

The blood was drawn through the right femoral artery, and the arterial blood gas was measured at certain time points (T0–T7). T0–T7 represent the following time points: the baseline, the end of ischemia (at 90 min after ischemia), and 30, 60, 90, 120, 180, and 240 min after reperfusion.

### Survival Analysis

After 4-h reperfusion, rats were extubated after recovery from anesthesia and observed for additional time to evaluate the survival rate. Rat death was identified by cessation of cardiac mechanical activity. The surviving rats were euthanized by overdosage of sodium pentobarbital after 168-h observation.

### Measurement of Lung H_2_S Level

The measurement of H_2_S production in lung tissues followed the established protocol (Sun et al., 2018). Briefly, lung tissues were homogenized in a 50 mM ice-cold potassium phosphate buffer (pH 6.8) containing a 100 mM potassium phosphate buffer (30 μl), 10 mM L-cysteine (20 μl), and 2 mM pyridoxyal 5’-phosphate (20 μl) and containing 1% zinc acetate (500 μl) as trapping solution. The reaction mixture was incubated at 37°C for 30 min. Subsequently, the reaction was stopped by 10% trichloroacetic acid (500 μl) followed by incubation with N,N-dimethyl-*p*-phenylenediamine sulfate (in 7.2 M HCl), which was followed by FeCl_3_ (30 mM, 400 μl) in 1.2 M HCl. After 20 min, the absorbance of the resulting solution at 670 nm was measured by spectrophotometer.

### SIRT1 Activity Measurements

SIRT1 activity was evaluated in tissue homogenates using a fluorometric assay (SIRT1 fluorogenic Assay Kit, BPS Bioscience, San Diego, CA). Acetylated p53 was the potential fluorophore substrate provided in the kit. After incubation with sirtuin-containing tissue homogenate, the substrate was deacetylated and was therefore sensitive to releasing green fluorophore when mixed with the color developer. Briefly, 20 μl of homogenated lung tissue was incubated with master mixture (5 μl diluted SIRT1 substrate, 0.5 μl NAD^+^, 5 μl BSA, and 14.5 μl SIRT assay buffer) for 30 min at 37°C. The fluorescence was detected by a fluorimeter with excitation set to 360 nm and emission set to 460 nm (Infinite M200 Pro, Tecan, Switzerland).

### Real-Time Quantitative PCR

Real-time PCR was performed as described previously (Jiang et al., 2019). Amplification was conducted using the following primers: 5′-TAGCCTTGTCAGATAAGGAAGGA-3′ (forward) and 5′-ACAGCTTCACAGTCAACTTTGT-3′ for rat SIRT1. The following thermal cycling protocol was used: 95°C for 5 min, followed by 40 cycles of amplification at 95°C for 10 s and 62°C for 20 s.

### Western Blot Analysis

Nuclear extracts were prepared using the Minute Cytoplasmic and Nuclear Fractionation Kit (Invent Biotechnologies Inc., Plymouth, MN). The nuclear fractionations were then subjected to Western blotting analysis using anti-Nrf2 antibody. Total proteins were subjected to Western blotting analysis using anti-SIRT1, anti-eNOS,anti-p-eNOS, and anti-HO-1 antibody. Western blot analysis was performed as previously described (Jiang et al., 2019).

### Immunohistochemical Analysis

Immunohistochemical staining was carried out as described previously (Jiang et al., 2019).

### Expression of eNOS Acetylation

The expression of eNOS acetylation was measured by co-immunoprecipitation assay, as previously described (Ding et al., 2015).

### Biotin Switch Assay

The assay was carried out as described previously (Du et al., 2019). Briefly, lung tissues were homogenized in RIPA lysis buffer. The primary anti-SIRT1 antibody was added into protein lysis containing Protein A beads (Sigma, St. Louis, MO, United States) and incubated overnight at 4°C. Beads were added to blocking buffer (HEN buffer with 2.5% SDS and 20 mM methyl methanethiosulfonate) at 50°C for 20 min. Methyl methanethiosulfonate was then removed by acetone, and the proteins were precipitated at −20°C for 20 min. After acetone removal, the proteins were resuspended in HENS buffer (HEN buffer with 1% SDS) and 4 mM biotin-HPDP. After incubation for 4 h at room temperature, biotinylated-protein was pulled down by streptavidin magnet beads and eluted by SDS-PAGE loading buffer and subjected to Western blot analysis.

### Assessment of Enzyme-Linked Immunosorbent

Serum concentrations of interleukin-6 and TNF-α were measured by enzyme-linked immunosorbent assay kits (R&D Systems, MN, United States) according to the manufacturer’s instructions.

### Determination of MPO, MDA, SOD, and T-AOC in Lung Tissue

As previously described, myeloperoxidase (MPO) activity, total antioxidative capability (T-AOC) levels, superoxide dismutase (SOD) activity, and malonaldehyde (MDA) activity were detected by commercial kits (Jiancheng Bio-Technology, Nanjing, China) (Jiang et al., 2019).

### Apoptosis Assay

Lung parenchymal cell apoptosis was detected by *In Situ* Cell Death Detection kit (Roche Molecular Biochemicals, Mannheim, Germany) as specified by the manufacturer. Cells with red nuclear staining were considered positive, as were all of the cells with DAPI (4′,6-diamidino-2-phenylindole) staining. The apoptotic index was expressed as the number of apoptotic nuclei/the total number of nuclei counted × 100%.

### Statistical Analysis

The data are expressed as mean ± standard deviation (SD). Statistical testing was performed using the Prism software package (version 5.0, GraphPad Software, La Jolla, CA, United States). Statistical significance was evaluated by one-way ANOVA followed by Tukey’s *post hoc* test for pairwise comparisons or a two-tail unpaired *t*-test. For survival analysis, the Kaplan–Meier method was used, followed by a log-rank (Mantel–Cox) test. A *P-*value of less than 0.05 was considered significant.

## Results

### Characterization of Diabetic Animals

**FIGURE 1:**
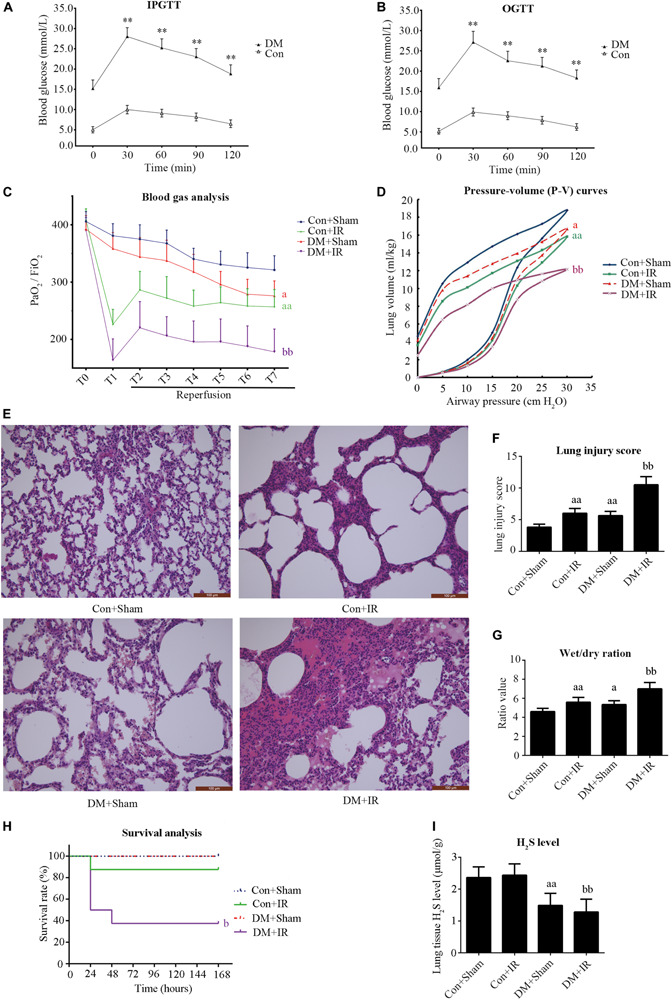
Type 2 diabetic rats subjected to lung IR injury exhibited significantly impaired lung function. **(A)** IPGTT, intraperitoneal glucose tolerance test. **(B)** OGTT, oral glucose tolerance test. **(C)** Arterial blood gas analysis. T0–T7 represent the following time points: baseline, end of ischemia, and 30, 60, 90, 120, 180, and 240 min after reperfusion. **(D)** Static compliance of the lung pressure–volume (P–V) curves. Data are represented by the mean values, and the bars are omitted for clarity. **(E)** Histologic analysis of lung tissues (magnification: 200×). **(F)** Lung injury score. **(G)** Wet/dry weight ratio. **(H)** Survival analysis. Rats were observed for 168 h (1 week), and survival time was calculated. **(I)** Lung H_2_S levels in rat. PaO_2_/FiO_2_: partial pressure of arterial oxygen (PaO_2_)/fraction of inspired oxygen (FiO_2_). IR, ischemia-reperfusion; DM, diabetes mellitus (**P < 0.01 versus Con group, ^*a*^*P* < 0.05 versus Con + sham group/^*aa*^*P* < 0.01 versus Con + sham group, ^b^*P* < 0.05 versus Con + IR group/^*bb*^*P* < 0.01 versus Con + IR group; *n* = 8 in each group).

As presented in Figures 1A,B and Table 1, compared with the non-diabetic rats, diabetic rats showed significantly impaired IPGTT and OGTT and increased blood glucose, serum total cholesterol, and total triacylglycerol, indicating that the type 2 diabetic model was successfully developed.

**TABLE 1:** Characterization of diabetic animals.

**Group**	**Con**	**DM**
Total cholesterol (mmol/L)	1.45 ± 0.16	4.61 ± 0.32**
Total triacylglycerol (mmol/L)	0.57 ± 0.07	1.43 ± 0.10**

DM: High-fat-diet-fed streptozotocin-induced type 2 diabetic group. Con: non-diabetic group. Results are expressed as mean ± SD. **P < 0.01 versus Con group; *n* = 8.

### Type 2 DM Aggravated Lung IR Injury

As presented in Figure 1C, there were no significant between-group differences with respect to the oxygenation index (PaO_2_/FiO_2_) at baseline. The PaO_2_/FiO_2_ (T7) values in the DM + Sham group were significantly lower than in the Con + Sham group (*P* < 0.05). The PaO_2_/FiO_2_ (T7) values in the DM + IR group showed a further decrease compared with the Con + IR group (*P* < 0.01). Similar tendencies were observed for the static compliance of the lung (the volume at a pressure of 30 cm H_2_O) (Figure 1D). The wet weight-to-dry weight ratio exhibited contrasting tendencies (Figure 1G).

As shown in Figures 1E,F, the lung morphologies in the Con + Sham groups remained intact. The DM + Sham group showed edema in the alveolar septa and spaces, interstitial thickening, intra-alveolar hemorrhage, and leukocyte infiltration, with higher lung injury scores (*P* < 0.01, compared with the Con + Sham group). These histological changes were more serious in the DM + IR group, with further increase in lung injury scores (*P* < 0.01, compared with the Con + IR group). Together, these experiments indicated the DM further aggravated lung injury in type 2 diabetic rats subjected to lung IR injury.

As shown in Figure 1H, the survival rate was significantly reduced in the DM + IR group compared with the Con + IR group (*P* < 0.05).

The endogenous H_2_S level of lung tissue in the DM + Sham group was decreased compared to the Con + Sham group, and the H_2_S level in the DM + IR group also showed a decrease compared with the Con + IR group (Figure 1I).

### Type 2 DM Impaired Lung SIRT1 Signaling and Aggravated Lung Oxidative Stress, Inflammation, and Apoptosis

**FIGURE 2:**
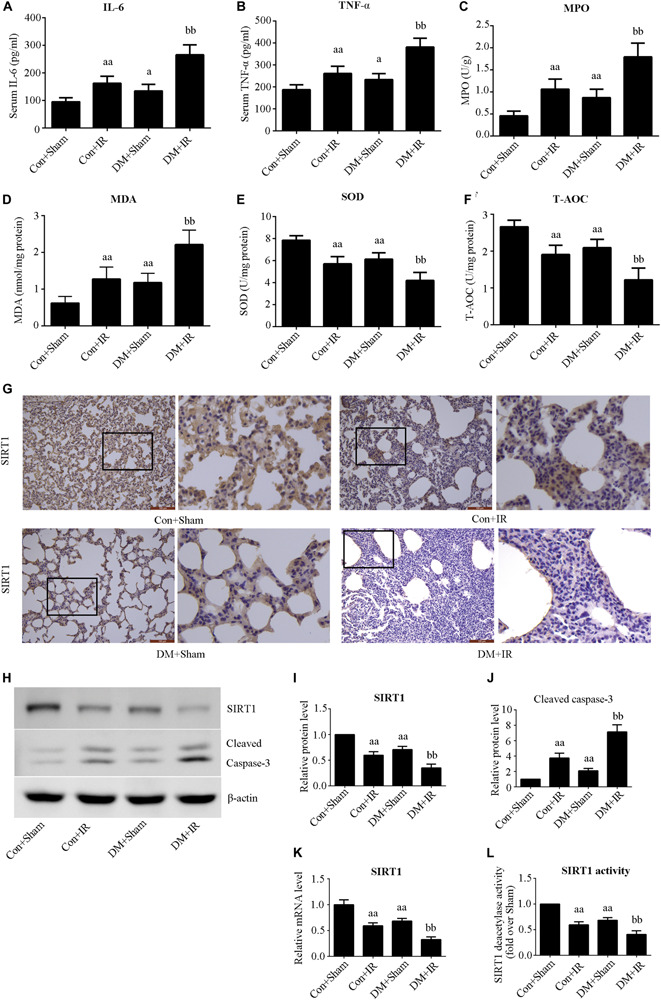
Type 2 DM impaired lung SIRT1 signaling and enhanced oxidative stress, inflammation, and apoptosis. **(A)** SOD concentration. **(B)** T-AOC concentration. **(C)** MDA concentration. **(D)** MPO concentration. **(E)** Serum concentrations of interleukin-6 (IL-6). **(F)** TNF-α. **(G)** Representative lung immunohistochemical images of SIRT1 (magnification: 200×). **(H)** Representative blots. **(I)** SIRT1 expression. **(J)** Cleaved caspase-3 expression. **(K)** mRNA expression of SIRT1 (*n* = 4). **(L)** Relative SIRT1 activity. IL, interleukin; TNF-a, tumor necrosis factor-a; MPO, myeloperoxidase; MDA, malonaldehyde; SOD, superoxide dismutase; T-AOC, total antioxidative capability (^*a*^*P* < 0.05 versus Con + sham group/^*aa*^*P* < 0.01 versus Con + sham group, ^bb^*P* < 0.01 versus Con + IR group; *n* = 8 in each group).

Figures 2A,B show that antioxidative capacity (activities of SOD and T-AOC) was lower in the DM + Sham group than in the Con + Sham group (*P* < 0.01), and these antioxidative-related indexes were further decreased in the DM + IR group (*P* < 0.01, compared with the Con + IR group). Contrasting tendencies were observed for MDA levels (Figure 2C), MPO activity (Figure 2D), serum concentrations of interleukin-6 (Figure 2E), and TNF-α (Figure 2F). Contrasting tendencies were also observed in the Western blot for cleaved caspase 3 (Figures 2H,J).

We measured the expression and activity of lung SIRT1 in both non-diabetic and diabetic rats. Figure 2 showed dramatically decreased SIRT1 expression and activity in type 2 DM rats (*P* < 0.01, the DM + Sham group compared with the Con + Sham group), which was further attenuated following lung IR injury (*P* < 0.01, the DM + IR group compared with the Con + IR group).

### EX527 Blunted H_2_S-Induced Pulmonary Protective Effect on Lung IR Injury in Type 2 Diabetic Rats

**FIGURE 3:**
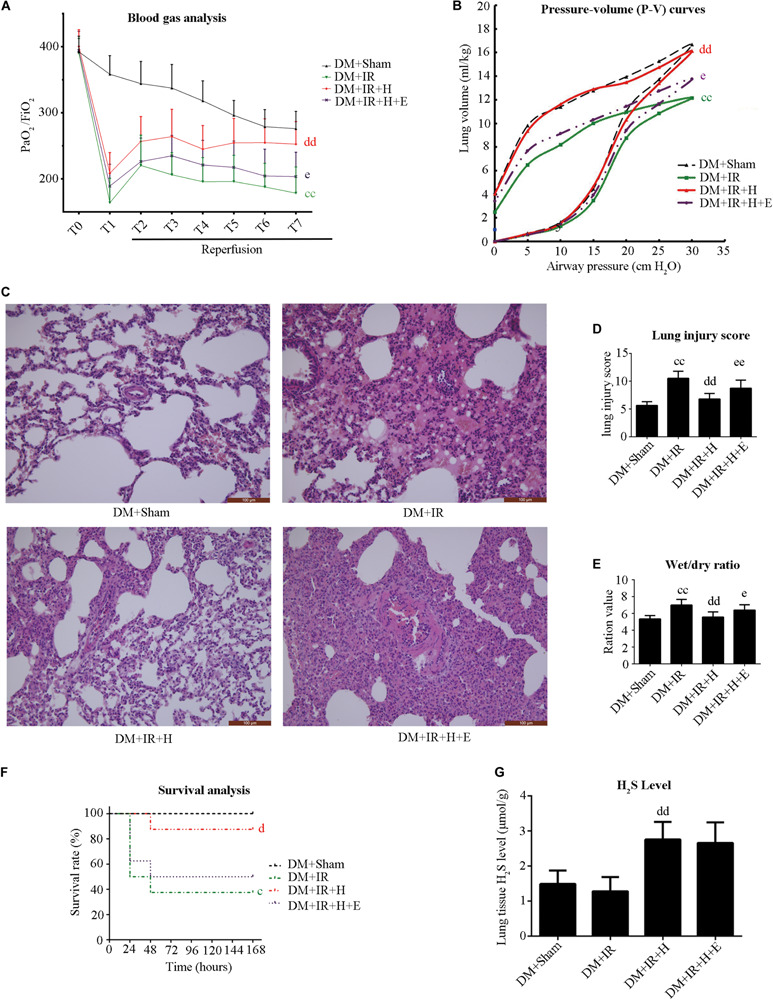
EX527 blunted GYY4137-induced pulmonary protective effect on lung IR injury in type 2 diabetic rats. **(A)** Arterial blood gas analysis. T0–T7 represent the following time points: baseline, end of ischemia, and 30, 60, 90, 120, 180, and 240 min after reperfusion. **(B)** Static compliance of the lung pressure–volume (P–V) curves. Data are represented by the mean values, and the bars are omitted for clarity. **(C)** Histologic analysis of lung tissues. (magnification: 200×). **(D)** Lung injury score. **(E)** Wet/dry weight ratio. **(F)** Survival analysis. Rats were observed for 168 h (1 week), and survival time was calculated. **(G)** Lung H_2_S levels in rat (^*c*^*P* < 0.05 versus DM + sham group/^*cc*^*P* < 0.01 versus DM + sham group, ^d^*P* < 0.05 versus DM + IR group/^*dd*^*P* < 0.01 versus DM + IR group, ^*e*^*P* < 0.05 versus DM + IR + H group/^*ee*^*P* < 0.01 versus DM + IR + H group; *n* = 8 in each group).

We used EX527 (the SIRT1-specific inhibitor) to unravel the role of H_2_S in diabetic lung IR injury as well as to elucidate the underlying mechanisms. Initially, we tested the toxic effect of EX527 administration. The experimental dosage of EX527 caused no significant changes to lung function (Supplementary Figure S1A–E) and apoptotic signaling (Supplementary Figure S1F,G) in diabetic rats (*P* > 0.05, the DM + IR + E group compared with the DM + IR group). As presented in Figure 3, H_2_S treatment significantly improved post-ischemic lung function in type 2 DM rats by increasing the oxygenation index (*P* < 0.01, the DM + IR + H group compared with the DM + IR group) and the static compliance of the lung (*P* < 0.01, the DM + IR + H group compared with the DM + IR group), decreasing the wet weight-to-dry weight ratio (*P* < 0.01, the DM + IR + H group compared with the DM + IR group). Meanwhile, H_2_S treatment markedly alleviated lung IR injury in diabetic rats, as evidenced by reduced lung injury scores (*P* < 0.01, the DM + IR + H group compared with the DM + IR group). However, these protective effects were abolished by EX527 administration (*P* < 0.05, the DM + IR + H + E group compared with the DM + IR + H group).

As shown in Figure 3F, H_2_S treatment improved the survival rate (*P* < 0.05, compared with the DM + IR group). However, survival did not differ between the DM + IR + H group and the DM + IR + H + E group (*P* = 0.0964). These data all suggested that SIRT1 played a pivotal role in the protective actions of H_2_S on diabetic lung IR injury.

Figure 3G shows that GYY4137 treatment recovered the reduction of endogenous H_2_S caused by diabetic lung IR injury (*P* < 0.01, the DM + IR + H group compared with the DM + IR group).

### EX527 Attenuated H_2_S-Induced Alleviation of the Inflammation in Diabetic Lung IR Injury

**FIGURE 4:**
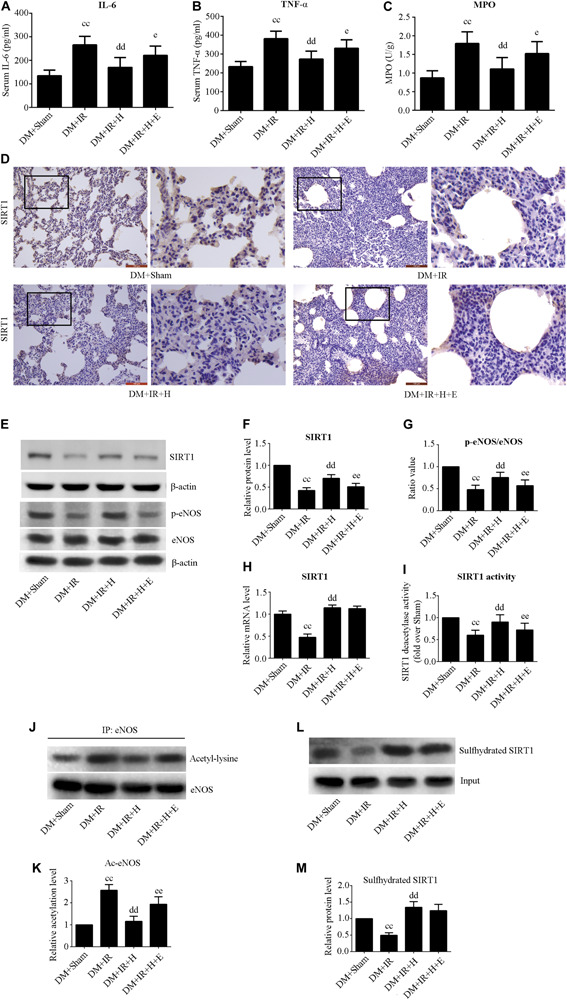
EX527 attenuated GYY4137-induced alleviation of the inflammation in diabetic lung IR injury. **(A)** Serum concentrations of interleukin-6 (IL-6). **(B)** TNF-α. **(C)** MPO concentration. **(D)** Representative lung immunohistochemical images of SIRT1 (magnification: 200×). **(E)** Representative blots. **(F)** SIRT1 expression. **(G)** The ratio of p-eNOS/eNOS. **(H)** mRNA expression of SIRT1 (*n* = 4). **(I)** Relative SIRT1 activity. **(J)** Representative blots. **(K)** Ac-eNOS. **(L)** Biotin switch assay. **(M)** Sulfhydrated SIRT1 expression. Ac-eNOS, acetylated endothelial nitric oxide synthase; eNOS, endothelial nitric oxide synthase; p-eNOS, phosphorylated endothelial nitric oxide synthase (^*cc*^*P* < 0.01 versus DM + sham group, ^dd^*P* < 0.01 versus DM + IR group, ^*e*^*P* < 0.05 versus DM + IR + H group/^*ee*^*P* < 0.01 versus DM + IR + H group; *n* = 8 in each group).

Figures 4A,B show that serum concentrations of interleukin-6 and TNF-α were obviously higher in the DM + IR group than in the DM + Sham group (*P* < 0.01). The serum levels of TNF-α and IL-6 in the DM + IR + H group were lower than those in the DM + IR group (*P* < 0.01). However, inhibition of SIRT1 markedly abolished the anti-inflammatory activity of H_2_S by increasing the serum level of TNF-α and IL-6 (*P* < 0.05, the DM + IR + H + E group compared with DM + IR + H group). Similar changes were observed in the MPO activity (Figure 4C). The expression levels of phosphorylated eNOS (activation state) showed a trend that was the inverse of that of the MPO activity (Figures 4E,G). Acetylated eNOS (deactivation state) showed the opposite changes compared with the previous levels (Figures 4J,K). These results all indicated that H_2_S reduced diabetic lung IR-induced inflammation and that SIRT1 mediated this action.

As showed in Figure 4, H_2_S treatment can rescue the impaired SIRT1 expression and activity caused by diabetic lung IR injury (*P* < 0.01, the DM + IR + H group compared with the DM + IR group). However, EX527 could abolish these effects (*P* < 0.01, the DM + IR + H + E group compared with the DM + IR + H group). The mRNA expression of SIRT1 was reduced in diabetic lung IR injury (*P* < 0.01, the DM + IR group compared with the DM + Sham group). The level of expression of SIRT1 mRNA was restored with GYY4137 treatment (*P* < 0.01, the DM + IR + H group compared with the DM + IR group). Sulfhydrated SIRT1 was upregulated by GYY4137 treatment (*P* < 0.01, the DM + IR + H group compared with the DM + IR group).

### EX527 Abolished H_2_S-Induced Suppression of the Oxidative Stress Level in Diabetic Lung IR Injury

**FIGURE 5:**
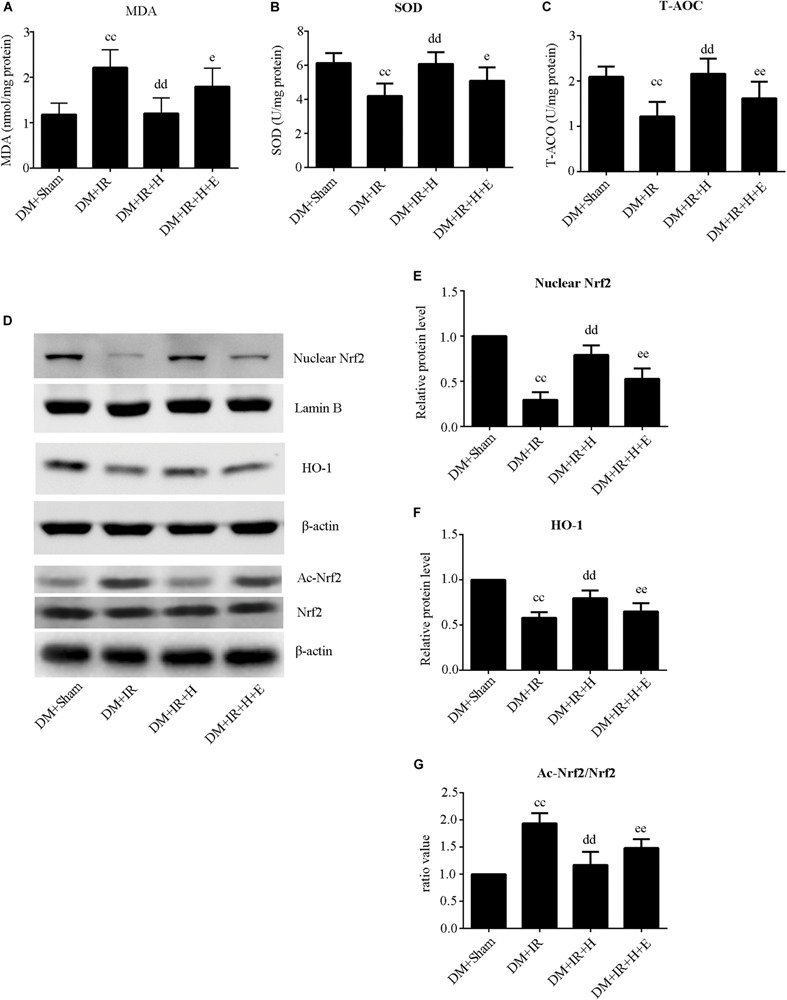
EX527 abolished GYY4137-induced suppression of the oxidative stress level in diabetic lung IR injury. **(A)** SOD concentration. **(B)** T-AOC concentration. **(C)** MDA concentration. **(D)** Representative blots. **(E)** Nuclear Nrf2 expression. **(F)** HO-1 expression. **(G)** The ratio of Ac-Nrf2/Nrf2. Ac-Nrf2, acetylated erythroid 2-related factor 2; Nrf2, Erythroid 2-related factor 2; HO-1, heme oxygenase 1 (^*cc*^*P* < 0.01 versus DM + sham group, ^dd^*P* < 0.01 versus DM + IR group, ^*e*^*P* < 0.05 versus DM + IR + H group/^*ee*^*P* < 0.01 versus DM + IR + H group; n = 8 in each group).

As shown in Figure 5A,B, activities of SOD and T-AOC were markedly lower in the DM + IR group than in the DM + Sham group (*P* < 0.01). Administration of GYY4137 improved antioxidative capacity by increasing the activities of SOD and T-AOC (*P* < 0.01, the DM + IR + H group compared with the DM + IR group). However, these antioxidative-related indexes were lower in the DM + IR + H + E group than in the DM + IR + H group (*P* < 0.05). The activities of MDA, an indicator of the oxidative stress response, showed contrasting tendencies (Figure 5C). Moreover, the expression levels of two antioxidative molecules, HO-1 and the nuclear accumulation of Nrf2, exhibited the same trend (Figures 5D–F). Contrasting tendencies were also observed in the Western blot for acetylation of Nrf2 (Figures 5D,G). Together, these experiments suggested that SIRT1 participated in the effect of H_2_S on antioxidative capacity and Nrf2/HO-1 signaling in diabetic lung IR injury.

### EX527 Compromised H_2_S-Induced Amelioration of the Cell Apoptosis in Diabetic Lung IR Injury

**FIGURE 6:**
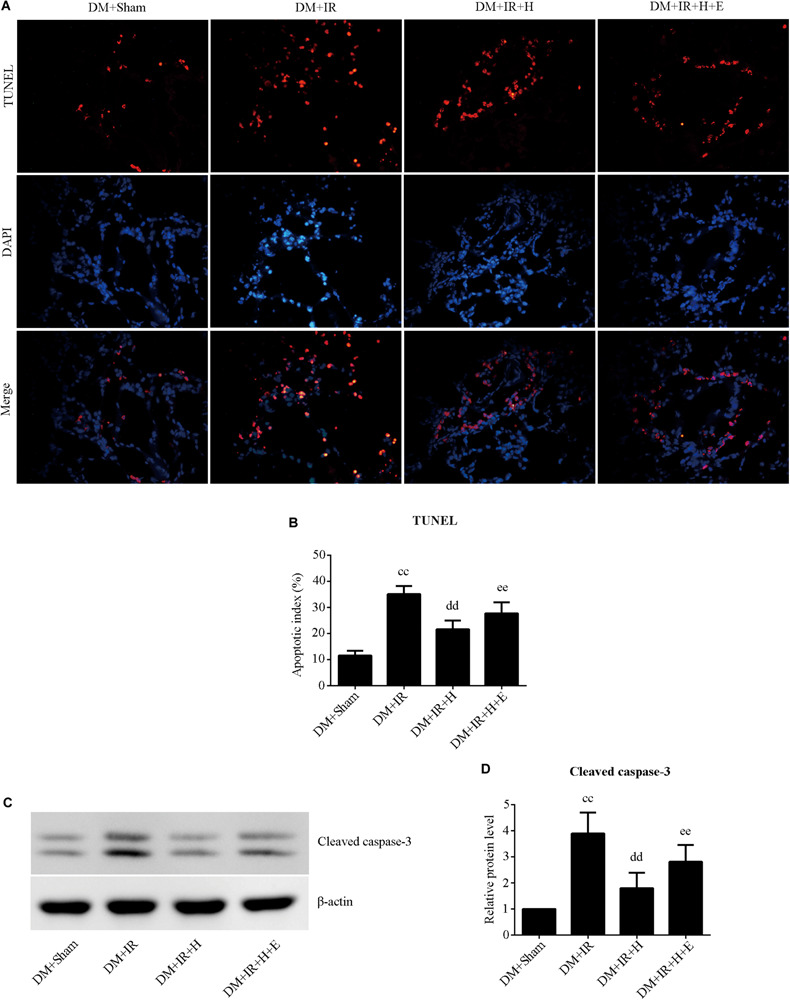
EX527 compromised H_2_S-induced amelioration of the cell apoptosis in diabetic lung IR injury. **(A)** Representative *in situ* detection of lung parenchymal cell apoptosis by TUNEL staining. (magnification: 400×). **(B)** Percentage of TUNEL-positive nuclei. **(C)** Representative blots. **(D)** Cleaved caspase-3 expression. TUNEL, Terminal deoxynucleotidyl transferase dUTP nick end-labeling (^*cc*^*P* < 0.01 versus DM + sham group, ^dd^*P* < 0.01 versus DM + IR group, ^*ee*^*P* < 0.01 versus DM + IR + H group; *n* = 8 in each group).

With respect to cell apoptosis, the apoptotic index (Figures 6A,B) was markedly increased in the DM + IR group compared with apoptosis levels in the DM + Sham group (*P* < 0.01). The number of apoptotic cells in the pulmonary parenchyma was significantly reduced in the DM + IR + H group compared with the DM + IR group (*P* < 0.01). However, the DM + IR + H + E group exhibited a markedly higher apoptotic index compared to the DM + IR + H group (*P* < 0.01). The expression levels of cleaved caspase 3 showed the same trend (Figures 6C,D). Together, these experiments indicated that the anti-apoptotic capability of H_2_S against lung IR injury was regulated by SIRT1 signaling in type 2 diabetic rats.

## Discussion

The major findings of the present study are as follows. (1) H_2_S effectively alleviated lung IR injury-induced oxidative stress, inflammation, and apoptosis in type 2 diabetic rats. (2) Lung SIRT1 signaling was markedly downregulated in type 2 diabetic rats, and it was further attenuated by IR injury. H_2_S administration effectively enhanced SIRT1 signaling in lung IR insult in type 2 diabetic rats. (3) Inhibition of SIRT1 abolished SIRT1-dependent Nrf2/HO-1 and eNOS activation, thus enhancing oxidative stress, inflammation, and apoptosis.

Type 2 DM is the most common form of diabetes, encompassing roughly 90% of diabetic patients (Chen et al., 2011). It has been well established that lung is a target of diabetic injury, although the lung is one of the least researched organs for diabetes complications (Wu et al., 2017). Recent studies have demonstrated that DM is a major risk factor for morbidity and mortality in lung transplant recipients (Hackman et al., 2014). Hyperglycemia upregulates poly ADP ribose polymerase (PARP), protein kinase C pathways, and the polyol pathway, which contribute to an NADH/NAD^+^ redox imbalance with increased levels of NADH as well as diminished levels of NAD^+^(Wu et al., 2017; Zheng et al., 2017). NADH/NAD^+^ redox imbalance contributes to reductive stress that gradually progresses to oxidative stress (Yan, 2014). Thus, under the diabetic condition, prolonged hyperglycemia leads to overproduction of ROS, which then enhance oxidative stress, inflammatory response, and cell apoptosis, which eventually aggravate reperfusion damage (Yu et al., 2015). We and others found that the diabetic state aggravated lung IR injury and that oxidative stress is a key factor in this process (Takahashi et al., 2016; Jiang et al., 2019). Consistent with these findings, we found markedly enhanced oxidative stress in diabetic rats following lung IR surgery in comparison to non-diabetic rats, which might lead to the increased inflammation and apoptosis, thus worsening physiological parameters of lung (oxygenation index, pulmonary compliance, and lung edema).

H_2_S is a novel endogenous signaling gasotransmitter affecting physiological and pathological processes of the respiratory system (Zhang et al., 2015). Due to its highly efficient antioxidative capacity, H_2_S has been demonstrated to play a potential role in preserving the function of and reducing insult to multiple organs, particularly induced by IR (Meng et al., 2017). Exogenous H_2_S could increase NAD^+^ levels to restore the ratio of NAD^+^/NADH and alleviate the redox imbalance resulting from diabetes (Sun et al., 2018). Under the diabetic condition, sustained hyperglycemia may cause tissues to increase their consumption of H_2_S, resulting in lower levels (Wallace and Wang, 2015). We have recently shown that H_2_S showed a protective role in diabetic lung IR by alleviating oxidative stress damage (Jiang et al., 2019). The results in our study demonstrated that H_2_S recovered the reduction of endogenous H_2_S caused by diabetic lung IR injury and protected against lung IR injury in type 2 diabetic rats. However, detailed information regarding how the signaling pathways mediate the protective effects of H_2_S against lung IR injury in the diabetic state remains largely unknown.

Silent information regulator 1 is a member of the class III group of histone deacetylase enzymes (Winnik et al., 2015). Silent information regulator 1 is proved to be a protective molecule against a wide variety of lung injuries, particularly that induced by IR (Liu et al., 2016). Silent information regulator 1 is sensitive to intracellular redox radicals and protects against oxidative stress in multiple systems (Alcendor et al., 2007). It has been shown that SIRT1 signaling protects against IR injury by upregulating antioxidants and downregulating oxidative stress as well as by decreasing pro-apoptotic molecules (Hsu et al., 2010). Here, we found that H_2_S effectively protected against diabetic lung IR injury by reducing oxidative damage in a SIRT1-dependent manner. Silent information regulator 1 also deacetylates NF-κB, hence effectively inhibiting its activity in *in vitro* and *in vivo* systems (Yeung et al., 2004). Nuclear factor-kappa B is a key regulator of multiple inflammatory pathways (Liu and Malik, 2006). A previous study demonstrated that SIRT1 participated in the anti-inflammatory activity in lipopolysaccharide-induced acute lung injury (Guo et al., 2015). ROS accumulation caused by diabetes contributes to the activation of nuclear transcription factors, such as NF-κB, which enhances inflammation and leads to endothelium dysfunction (Evans et al., 2002; Donath and Shoelson, 2011). In this study, we observed that SIRT1-mediated suppression of inflammation played a critical role in the protective activity of H_2_S in diabetic lung IR insult. Numerous studies found that SIRT1 expression was significantly decreased in the diabetic state and that overexpression of SIRT1 produced beneficial effects on glucose homeostasis (Milne et al., 2007). SIRT1 has been emerging as an effective therapeutic target for the treatment of type 2 DM by regulating lipid mobilization and adiponectin excretion (Picard et al., 2004), controlling fatty acid oxidation and mitochondrial biogenesis (Gerhart-Hines et al., 2007), protecting pancreatic β-cells (Lee et al., 2009), and modulating insulin secretion (Bordone et al., 2006). Several lines of evidence indicate that SIRT1 is a promising protector against IR-induced tissue injury under diabetic conditions (Ding et al., 2015; Yu et al., 2015, 2017). In addition, H_2_S increased intracellular NAD^+^ levels, an effect that was SIRT1-dependent (Das et al., 2018). Therefore, H_2_S increases SIRT1 expression and protects against oxidative stress (Suo et al., 2013; Wu et al., 2015). Zinc ions play a key role in SIRT1 function, and Zn^2+^ binding to the zinc-finger motif is essential for the deacetylase activity of SIRT1 and its structural integrity (Chen et al., 2010). Sulfhydration promotes Zn^2+^-tetrathiolate, stabilizes the alpha-helix, and thus enhances the deacetylase activity. H_2_S directly sulfhydrated SIRT1 and enhanced SIRT1 binding to zinc ion, which promoted SIRT1 deacetylation activity (Du et al., 2019). Consistent with these studies, we observed that H_2_S treatment can rescue the impaired sulfhydrated SIRT1 caused by diabetic lung IR injury. Here we also found that lung SIRT1 expression and activity is significantly downregulated in type 2 diabetic rats and that reperfusion injury further aggravated these effects. H_2_S enhanced SIRT1 expression and activity, whereas these effects were largely attenuated by SIRT1 inhibitor. Furthermore, inhibition of SIRT1 signaling largely abolished the protective actions of H_2_S, suggesting that SIRT1-mediated reducing oxidative stress, inflammation, and apoptosis played a critical role in the protective actions of H_2_S.

Another major finding of this study is that H_2_S activated Nrf2/HO-1 signaling and phosphorylation of eNOS (activation of eNOS). Treatment with EX527 largely abolished these effects of H_2_S. Nrf2, as an important regulator of cellular antioxidant defense mechanisms, plays a protective role against lung injury (Meng et al., 2016). Once activated, Nrf2 translocates to the nucleus, where it transcribes several antioxidant genes, such as heme oxygenase 1 (HO-1), which maintains redox homeostasis and regulates the inflammatory response (Chapple et al., 2012). It has also been demonstrated that enhanced HO-1 expression could attenuate lung IR injury by alleviating oxidative stress and inflammation (Sun et al., 2017b). Recent research showed that Nrf2/HO-1 signaling plays a critical role in reducing IR injury, especially in diabetic setting (Peake et al., 2013). In addition, impaired Nrf2/HO-1 signaling was a key contributor to aggravated IR injury in diabetic animals (Gao et al., 2016). In this study, we found that GYY4137 treatment significantly increased nuclear Nrf2 expression and enhanced Nrf2-dependent HO-1 transcription in diabetic lung IR injury. Conversely, these effects were abrogated by SIRT1 inhibition. These data suggest that SIRT1 might act as upstream regulatory signal transduction of Nrf2/HO-1. In fact, our data are consistent with the previous report that SIRT1 modulates the transcription factor Nrf2 in regulating the transcription of gene encoding antioxidant enzymes to resist oxidative damage (Zhang et al., 2018). Recent studies have also demonstrated that SIRT1 deacetylated Nrf2, thus increasing the activity and stability of Nrf2, resulting in resistance to oxidative stress and suppression of apoptosis (Ding et al., 2016). Here, we found that GYY4137 treatment rescued the increased acetylation of Nrf2 caused by diabetic lung IR injury, whereas these effects were largely attenuated by SIRT1 inhibitor. Therefore, we concluded that GYY4137 might increase Nrf2/HO-1 signaling through activating SIRT1. Endothelial nitric oxide synthase can maintain endothelial barrier properties and prevent leukocyte infiltration (Kaminski et al., 2007). Indeed, activation of eNOS has been demonstrated as a key factor that protects against lung IR injury (Matsuo et al., 2015). It is known that diabetes can cause significant endothelial dysfunction due to decreased NO bioavailability. Oxidative stress is a critical driver of impaired endothelial NO bioavailability in the diabetic state, which participates in the pathogenesis and progression of diabetic tissue injury (Suganthalakshmi et al., 2006). Decreased expression of eNOS and impaired NO synthesis have been detected in both type 2 DM patients and diabetic animals, which contributes to the pathophysiology of diabetes (Torres et al., 2004; Ding et al., 2015). SIRT1 colocalizes with eNOS, and deacetylation by SIRT1 increases eNOS activity. Inhibition of SIRT1 activity can acetylate eNOS and decrease eNOS activity (Mattagajasingh et al., 2007). Our data showed that GYY4137 enhanced eNOS phosphorylation (activation state) and reduced eNOS acetylation (deactivation state) while SIRT1 inhibition markedly reduced the effect of GYY4137 on the activity of eNOS. All of these data indicated that GYY4137 might enhance Nrf-2/HO-1 signaling and maintain normal eNOS function and active form via SIRT1 activation, thus reducing oxidative stress, inflammation, and apoptosis in diabetic lung IR injury.

This study has several limitations. First, we used a high-fat-diet-fed streptozotocin-induced type 2 diabetic model to simulate the clinical presentations of type 2 DM patients, but whether this model completely substitutes for diabetes requires further detailed elucidation. Second, our animal experiments were exclusively on a warm IR model without transplantation, but whether the study findings can be performed in a cold IR model with transplantation needs further investigation. Third, the finding that H_2_S upregulated SIRT1-mediated protective effects in diabetic lung by enhancing Nrf2/HO-1 signaling and maintaining normal eNOS function was verified by using SIRT1 inhibitor EX527 rather than by genetic manipulation.

## Conclusion

The present study indicates that lung SIRT1 signaling was significantly downregulated in type 2 diabetic rats, and it was further attenuated by IR injury. Exogenous H_2_S treatment significantly alleviated IR-induced lung functional dysfunction, oxidative stress, inflammation, and apoptosis by SIRT1 signaling reactivation in type 2 diabetic rats. This study provides a novel theoretical basis for the development of new therapeutic strategies to treat type 2 diabetic patients with ischemic lung disease.

## Data Availability Statement

All datasets generated for this study are included in the article/Supplementary Material.

## Ethics Statement

The animal study was reviewed and approved by Institutional Animal Care and Use Committee at Harbin Medical University.

## Author Contributions

TJ contributed to conception of the work. TJ, ZS, TL, and XL performed the experiments and collected the data. WY and HZ contributed to analysis and interpretation of data for the work. TJ and WY drafted the manuscript. All authors reviewed the manuscript.

## Conflict of Interest

The authors declare that the research was conducted in the absence of any commercial or financial relationships that could be construed as a potential conflict of interest.

## Supplementary Material

FIGURE S1 The effect of EX527 on diabetic lung. **(A)** Arterial blood gas analysis. T0-T7 represent the following time points: baseline, end of ischemia, and 30 min, 60 min, 90 min, 120 min, 180 min, and 240 min after reperfusion. **(B)** Static compliance of the lung pressure–volume (P–V) curves. Data are represented by the mean values, and the bars are omitted for clarity. **(C)** Histologic analysis of lung tissues. (magnification: 200 ×). **(D)** Lung injury score. **(E)** Wet/dry weight ratio. **(F)** Representative blots. **(G)** Cleaved caspase-3 expression (n = 8 in each group).
