# Supplementary figures and images for: Hydrogen Sulfide Ameliorates Lung Ischemia-Reperfusion Injury Through SIRT1 Signaling Pathway in Type 2 Diabetic Rats
Source: Front Physiol. 2020 Jun 30;11:596. doi: 10.3389/fphys.2020.00596 (PMC7338566; doi:10.3389/fphys.2020.00596)

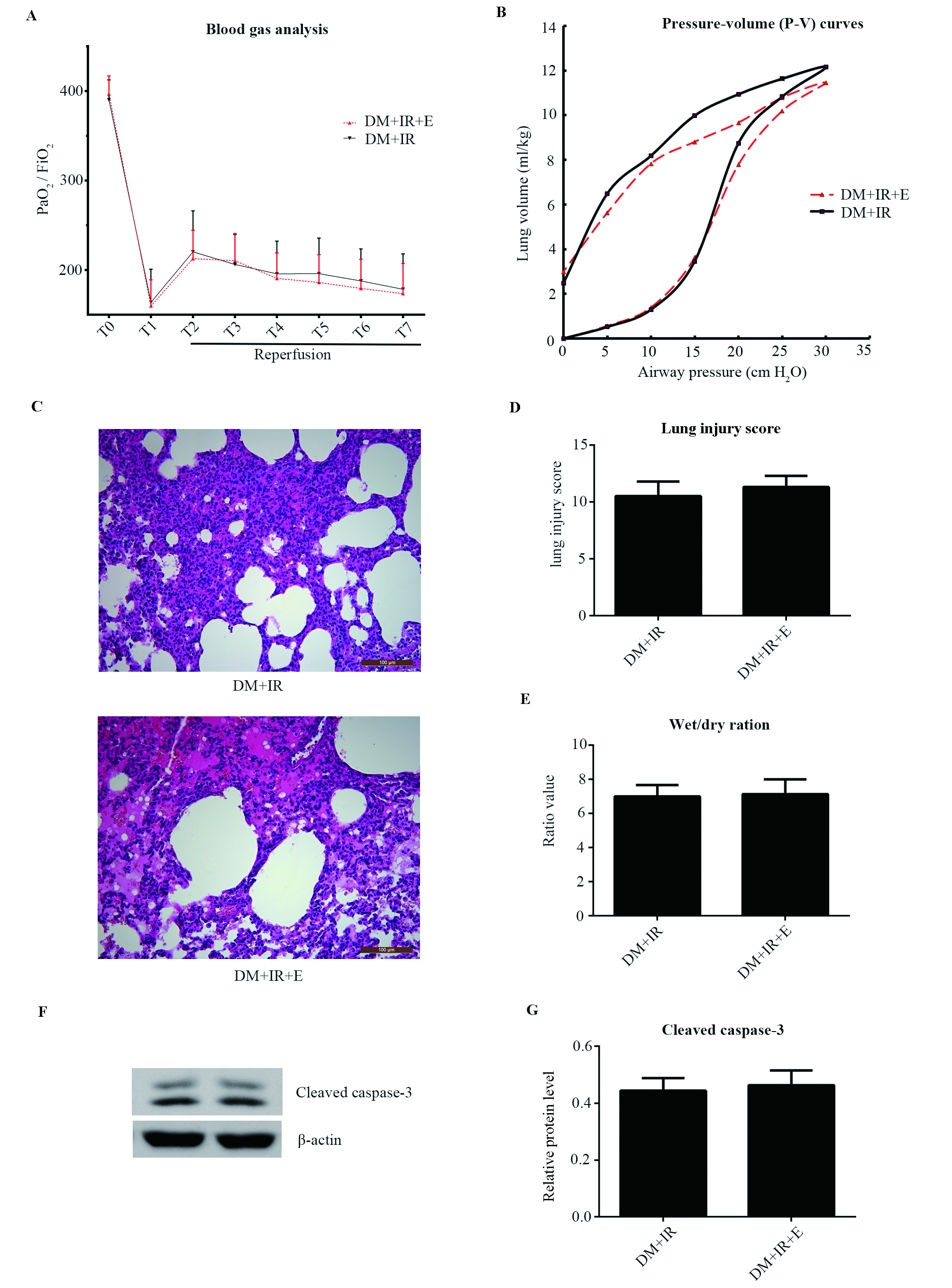

Supplement: FIGURE S1 — The effect of EX527 on diabetic lung. (A) Arterial blood gas analysis. T0-T7 represent the following time points: baseline, end of ischemia, and 30 min, 60 min, 90 min, 120 min, 180 min, and 240 min after reperfusion. (B) Static compliance of the lung pressure–volume (P–V) curves. Data are represented by the mean values, and the bars are omitted for clarity. (C) Histologic analysis of lung tissues. (magnification: 200 ×). (D) Lung injury score. (E) Wet/dry weight ratio. (F) Representative blots. (G) Cleaved caspase-3 expression (n = 8 in each group). [file Image_1.TIF]
